# Biocompatible and Antibacterial Nitric Oxide-Releasing Pluronic F-127/Chitosan Hydrogel for Topical Applications

**DOI:** 10.3390/polym10040452

**Published:** 2018-04-18

**Authors:** Milena T. Pelegrino, Bruna de Araujo Lima, Mônica H. M. do Nascimento, Christiane B. Lombello, Marcelo Brocchi, Amedea B. Seabra

**Affiliations:** 1 Center for Natural and Human Sciences, Universidade Federal do ABC, Av. dos Estados 5001, Santo André, SP, CEP 09210-580, Brazil; pelegrino.milena@ufabc.edu.br (M.T.P.); Christiane.lombello@ufabc.edu.br (C.B.L.); 2 Nanomedicine Research Unit (NANOMED), Universidade Federal do ABC, Av. dos Estados 5001, Santo André, SP 09210-580, Brazil; monica.nasci@hotmail.com; 3 Tropical Disease Laboratory, Department of Genetics, Evolution, Microbiology and Immunology, Institute of Biology, University of Campinas, Campinas, SP 13083-862, Brazil; brunalimabio@gmail.com (B.d.A.L.); mbrocchi@unicamp.br (M.B.); 4 Center for Engineering, Modeling and Applied Social Science, Universidade Federal do ABC, Alameda da Universidade sem numero, São Bernardo do Campo, SP, CEP 09606-045, Brazil

**Keywords:** chitosan, thermoresponsive hydrogel, nitric oxide, *S*-nitrosothiols, biocompatibility, antimicrobial, *Pseudomonas aeruginosa*, Pluronic F127

## Abstract

Nitric oxide (NO) is involved in physiological processes, including vasodilatation, wound healing and antibacterial activities. As NO is a free radical, designing drugs to generate therapeutic amounts of NO in controlled spatial and time manners is still a challenge. In this study, the NO donor *S*-nitrosoglutathione (GSNO) was incorporated into the thermoresponsive Pluronic F-127 (PL)-chitosan (CS) hydrogel, with an easy and economically feasible methodology. CS is a polysaccharide with known antimicrobial properties. Scanning electron microscopy, rheology and differential scanning calorimetry techniques were used for hydrogel characterization. The results demonstrated that the hydrogel has a smooth surface, thermoresponsive behavior and good mechanical stability. The kinetics of NO release and GSNO diffusion from GSNO-containing PL/CS hydrogel demonstrated a sustained NO/GSNO release, in concentrations suitable for biomedical applications. The GSNO-PL/CS hydrogel demonstrated a concentration-dependent toxicity to Vero cells, and antimicrobial activity to *Pseudomonas aeruginosa* (minimum inhibitory concentration and minimum bactericidal concentration values of 0.5 µg·mL^−1^ of hydrogel, which corresponds to 1 mmol·L^−1^ of GSNO). Interestingly, the concentration range in which the NO-releasing hydrogel demonstrated an antibacterial effect was not found to be toxic to the Vero mammalian cell. Thus, the GSNO-PL/CS hydrogel is a suitable biomaterial for topical NO delivery applications.

## 1. Introduction

Nitric oxide (NO) is an endogenous molecule involved in several biological signaling pathways. At low concentrations (pico- to nanomolar), NO has regulatory functions, such as the promotion of vasodilation, wound healing, cell proliferation, inhibition of platelet adhesion and aggregation [1,2,3]. At higher concentrations (micro- to millimolar), NO has antitumor and antimicrobial effects [2,3,4]. NO is considered a promising alternative to conventional antimicrobials. However, it is a challenge to deliver a precise NO concentration directly to the target site of application, in sustained and convenient manners and with minimal side effects [1].

NO is a free radical (half-life one to five seconds), and can be inactivated in biological systems upon reaction with biomolecules (such as hemoglobin), thus limiting its effects. To overcome this issue, several NO-releasing biomaterials have been prepared to carry and deliver therapeutic amounts of NO for different biomedical applications [4]. In topical applications, NO-releasing biomaterials can be used for coating medical devices [5]. In dermatological applications, it can be used to promote dermal blood flow [6,7,8,9], for antimicrobial effects [2,10,11], cell proliferation [4], wound repair [12,13,14,15], controlling inflammatory pain [16] and local warming [17]. In addition, administration of NO-releasing materials on human skin might have a therapeutic effect against endothelial dysfunctions, such as vasoconstriction, vasospasm and thrombosis [5,16,18].

**Figure 1:**
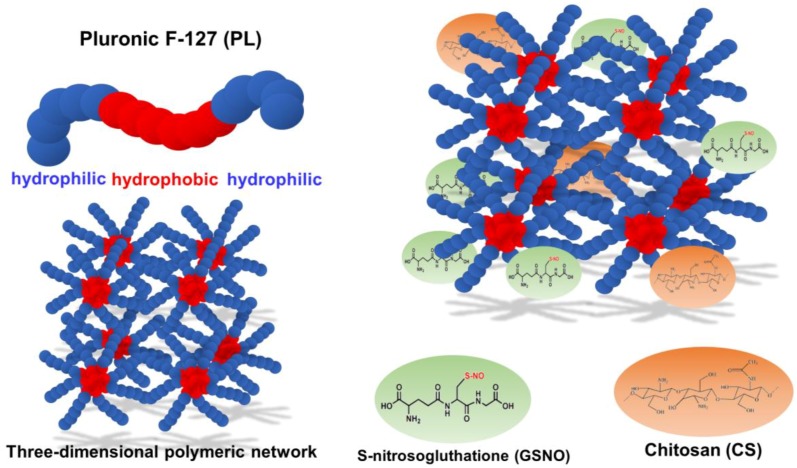
Schematic representation of the formation of three-dimensional polymeric network of Pluronic F-127 (PL) hydrogel structure containing chitosan (CS) and *S*-nitrosoglutathione (GSNO) to delivery NO.

In this context, there is great interest in the development of NO-releasing biomaterials capable of releasing NO/NO donors in a controllable manner for topical applications. In this study, the NO donor *S*-nitrosoglutathione (GSNO) was incorporated into a thermoresponsive hydrogel comprised of poly(ethylene oxide)-poly(propylene oxide)-poly(ethylene oxide), PEO-PPO-PEO (Pluronic F127) and chitosan (CS) (Figure 1).

GSNO is an endogenous molecule that spontaneously releases free NO [1,2]. Hydrogels are soft materials with a three-dimensional network and viscoelastic properties. Thermoresponsive hydrogels can change their rheological properties with changes in temperature [19]. Pluronic (PL) is approved for human uses by the Food and Drug Administration (FDA), and has been extensively employed as a drug carrier and for tissue engineering [20]. Pluronics (Sigma) or Poloxamer (Bayer) are co-polymers largely used for hydrogel preparation. They are made of poly(ethylene oxide)-poly (propylene oxide)-poly(ethylene oxide) (PEO-PPO-PEO) units. PPO segments are more hydrophobic, whereas PEO is more hydrophilic [21,22,23]. There are several types of Pluronics, depending on the number of PEO and PPO units [23]. In this study, we used the Pluronic F-127 (PL) with 106 units of PEO and 70 units of PPO [23]. Pluronic has also been found to decrease the viscosity of the blood, prevent aggregation of erythrocytes and adhesion to the vascular endothelium layer [21]. In aqueous environments, PL chains undergo a spontaneous self-organization due to the differences is solubility between PEO and PPO units, leading to the formation of micelles (micellization process) [22,23,24]. The PPO segment of PL is comprised of the hydrophobic core of the micelles, whereas the PEO segment of PL is comprised of a hydrophilic shell [21]. The gelation process determines the space organization of the micelles (hexagonal, cubic or lamellar phases) [23]. Upon an increase in the temperature and/or PL concentration, micelles auto assemble, leading to a three-dimensional polymeric network, as illustrated in Figure 1 [23]. The gelation process leads to an increase in the viscosity of the polymer solution, leading to a gel state [23]. The Pluronic systems are thermosensitive, which means they exist as liquids at low temperatures and become semi-solid at higher temperatures. This phenome is reversible [21,23]. This feature is suitable for pharmacological formulations, since it is possible to incorporate active drugs at low temperature, and the increase in the temperature leads to the formation of a hydrogel containing the drug. The main advantage of PL hydrogels is the ease of preparation, that does not require the use organic solvents [21].

The polysaccharide chitosan (CS) was incorporated into PL hydrogel. CS is biodegradable, biocompatible, non-allergenic and an inexpensive biological polymer extensively used for tissue engineering and drug delivery [25,26,27]. Chitosan is obtained from chitin, which is the major component in crustacean shells, such as crabs and insects [26], so its use in technological products is of ecological and sustainable importance [28]. In addition, CS has important effects, such as antimicrobial, anticholesterolemic, antitumor, hemostatic and antioxidant activities [25,26,27].

In this present work, an NO-releasing hydrogel was prepared by incorporating the NO donor (GSNO) into a PL/CS hydrogel. Both PL and CS are biocompatible polymers extensively employed in medical applications. PL is responsive to forming the hydrogel network. This matrix permits an easy topical application allowing a site-specific release of the NO donor/NO. The presence of CS into the hydrogel matrix provides advantages due to its muco-adhesiveness characteristic over commercial formulations [25,26,27]. In biological systems, at low concentrations, GSNO has regulatory effects (promotion of tissue regeneration, wound healing and vasodilation), whereas at high concentrations it has antimicrobial effects [3]. Therefore, all of these combined properties can compose a versatile and suitable material for drug delivery of NO in topical applications. To the best of our knowledge, this is the first paper to describe the preparation and characterization of NO-releasing PL/CS hydrogel with biocompatibility and antibacterial effect. Our results demonstrated a sustained NO/NO donor release from the hydrogel matrix, at therapeutic levels. The combination of NO and CS demonstrated a synergistic effect against *Pseudomonas aeruginosa.* Interestingly, the concentration of NO-releasing hydrogel required for an antibacterial effect was not toxic towards mammalian cells, indicating the promising and safe uses of this biomaterial in topical applications.

## 2. Materials and Methods

### 2.1. Materials

Chitosan (CS, low molecular weight, 75% deacetylation), glutathione (GSH), sodium nitrite (NaNO_2_), Pluronic F-127 (PL) ([poly(ethylene oxide)]_106_[poly(propylene oxide)]_70_[poly(ethylene oxide)]_106_, molar weight of 12,600 kDa), phosphate buffer saline (PBS, pH 7.4), MTT (3-(4,5-dimethylthiazol-2-yl)-2,5-diphenyltetrazolium bromide) and phenol were obtained from Sigma-Aldrich (St. Louis, MO, USA). Acetone, hydrochloric acid and lactic acid were obtained from Labsynth (Diadema, SP, Brazil). All experiments were carried out using analytical grade water from a Millipore Milli-Q Gradient filtration system (resistivity below 18.2 MΩ·cm^−1^ at 25.0 °C). The Vero cell line was obtained from Instituto Adolfo Lutz (São Paulo, SP, Brazil). The culture medium, supplements, antibiotics and bovine fetal serum were obtained from Cultilab (Campinas, SP, Brazil). The Mueller-Hinton media (broth and agar) were purchased from Difco (Franklin Lakes, NJ, USA), and the sodium chloride (NaCl, P.A. grade), used to obtain saline solution 0.85% *w*/*v*, was purchased from J.T. Baker (Ciudad del Mexico, Mexico).

### 2.2. Preparation of PL/CS Hydrogel

PL/CS hydrogel was prepared based on the cold method first described by Schmolka et al. [29]. This method has been reported extensively for the preparation of PL-based hydrogels containing drugs, particles or other polymers [27,30,31,32]. First, 0.1 g of CS was dissolved in 10 mL of lactic acid (2%) under magnetic stirring at room temperature. Next, 2.0 g of PL was gradually added to the CS solution under gentle magnetic stirring. The temperature was maintained at 4 °C until complete dissolution of PL powder [20]. The final concentrations of PL and CS in the hydrogel were 20% *w*/*v* and 1% *w*/*v*, respectively. This material is referred as PL/CS hydrogel.

### 2.3. Synthesis of GSNO

GSNO was synthesized as previously described [33,34,35]. GSH (1228 mol·L^−1^) was dissolved into HCl (1 mol·L^−1^), followed by the addition of an equimolar amount of NaNO_2_, correlated to GSH. The nitrosation reaction was carried out for 20–30 min, in an ice bath, under magnetic stirring and in the dark, leading to the formation of a pink precipitate of GSNO. This material was washed with cold water and filtered under vacuum using a Millipore cellulose membrane. The obtained GSNO was freeze-dried for 24 h. Solid GSNO was maintained in a recipient with humidity controlled at −20 °C [22].

### 2.4. Incorporation of GSH or GSNO into PL/CS Hydrogel

GSH or GSNO were incorporated into the PL/CS hydrogel matrix following the process described by Pelegrino et al. with modifications [33]. The required amount of powdered GSH or GSNO was added to the PL/CS solution, under gentle magnetic stirring and light protection, at 5 °C. This mixture was maintained at 5 °C until the complete dissolution of the GSH or GSNO. This process led to the formation of a PL/CS hydrogel, at room temperature (final concentrations of PL and CS were 20% and 1% of CS *w*/*v*, respectively) containing 50 mmol·L^−1^ of GSH or GSNO (final concentration). This is henceforth referred to as GSH-PL/CS hydrogel or GSNO-PL/CS hydrogel, respectively.

### 2.5. Morphological Characterization of PL/CS and GSH-PL/CS Hydrogels

The morphology of the PL/CS and GSH-PL/CS hydrogels was investigated by scanning electron microscopy (SEM). Prior the analysis, the samples were frozen at −80 °C for 12 h and freeze-dried for 36 h (Labconco, 84C, Kansas City, MO, USA), as previously described [24,36,37,38]. The dried materials were spread on a double-sided conduction adhesive tape, pasted on a metallic stub and analyzed using a benchtop SEM (JCM-6000PLUS NeoScope, JEOL Technics, Tokyo, Japan). The analysis was done under high vacuum and at an operating voltage of 5 kV.

### 2.6. Rheological Measurements of PL/CS and GSH-PL/CS Hydrogels

Rheological measurements of PL/CS and GSH-PL/CS hydrogels were performed on a rotational rheometer at oscillation modus (Kinexus Lab, Malvern Instruments Ltd., Worcestershire, UK) with a parallel plate (PP) geometry (diameter 70 mm, angle 2.576 rad and gap between the plates of 1.02 mm) and a Peltier temperature-controlled holder. For determination of elastic (*G*′) and loss (*G*″) moduli, a volume of 1 mL of each hydrogel sample were transferred to the rheometer. A temperature ramp was applied from 10 to 50 °C at a heating rate of 5.0 °C·min^−1^, with a fixed frequency of 1.0 Hz and a shear stress of 2.0 pA. The temperature when *G*′ > *G*″, and the sample demonstrated a solid-like gel formation, was related to gelling temperature (*T*_gel_). Each experiment was repeated twice and analyzed using rSpace software (software version 2.00, Worcestershire, UK). In a further step, the temperature was fixed at 32.5 °C and the frequency was varied from 0.1 to 10 Hz in order to evaluate the stability of the hydrogel. Each experiment was repeated twice and analyzed using rSpace software.

### 2.7. Thermal Analyses of PL/CS and GSH-PL/CS Hydrogels

Thermal characterization of the PL/CS and GSH-PL/CS hydrogels was performed by Differential Scanning Calorimetry (DSC) employing the DSC Q200 equipment (TA Instruments, New Castle, DE, USA). The reference in all cases was a standard aluminum sample holder. Samples of 0.2 mL of PL/CS or GSH-PL/CS hydrogel were analyzed using heating–cooling cycles in the range of 0–65 °C, with a heating rate of 5 °C·min^−1^, employing nitrogen to purge the gas chamber (50 mL·min^−1^). The cycles were as follows: cycle 1 cooled from 25 to −10 °C, cycle 2 heated from −15 to 65 °C, cycle 3 cooled from 65 to −15 °C and cycle 4 heated from −15 °C to 65 °C. The data were analyzed using Universal Analysis 2000 software (TA Instruments version 4.5 A, New Castle, DE, USA). The critical micellization temperature (CMT) was determined from the endothermic peak in the recorded DSC thermographs, and the enthalpy change (∆*H*) of the endothermic peak was calculated.

### 2.8. Kinetics of NO Release

Kinetics of NO release from aqueous GSNO and from GSNO-containing hydrogel (GSNO-PL/CS) were monitored by measuring changes in absorbance intensity at 545 nm (nN→π* transition), by using the UV–visible spectrophotometer (Agilent 8454, Palo Alto, CA, USA), with a temperature-controlled sample holder. Changes at 545 nm are solely associated with S–N bond cleavage and free NO release [33,34,39]. Kinetic data were acquired at temperatures of 25, 32.5 and 37 °C. The initial concentration of GSNO in all groups was 50 mmol·L^−1^, as confirmed by the detection of the characteristic GSNO absorption band at 545 nm (nN→π*) [33,39]. The quantity of NO released over time was calculated following Equations (1) and (2) [22,40]. The results were reported as the mean ± standard deviation (SD) of three independent experiments and expressed in terms of the NO concentration:

[NO]t = [GSNO]_0_ − [GSNO]_t_, (1)

(2) $$[\mathrm{NO}]t\;=\;\frac{A_{0}b}{\varepsilon \mathrm{GSNO}}\;-\;\frac{A_{t}b}{\varepsilon \mathrm{GSNO}}.$$

Equations (1) and (2) relate NO concentration at time, *t*, ([NO]t) to the GSNO absorption band, wherein [GSNO]_0_ and [GSNO]_t_ are the concentrations of GSNO at the beginning of the reaction and at time, *t*, respectively. Variables *A*_0_ and *A*_t_ are the GSNO absorbances, at 545 nm, at the beginning of the monitoring and at time, *t*, respectively. Variable εGSNO is the molar absorption coefficient of GSNO at 545 nm (ε equals to 18.4 mol^−1^·L·cm^−1^), and b is the optical path of cuvette, which corresponds to 1 cm.

### 2.9. In Vitro GSNO Diffusion

The in vitro kinetics of intact GSNO diffusion from aqueous GSNO and from GSNO-containing hydrogel (GSNO-PL/CS) were carried out by using a vertical diffusion cell (standard cell, 15 mm of diameter, 7 mL, Hanson Research Corporation) [41]. The cell consisted of donor and receptor chambers, separated by polysulfone membrane disc filters with a 450 nm pore size (Tuffryn, Pall Corporation, Port Washington, NY, USA). The donor compartment was filled with 2.5 mL aqueous GSNO (GSNO in PBS) or GSNO-PL/CS hydrogel, in which the initial GSNO concentration was 50 mmol·L^−1^, in both cases. The receptor compartment was filled with PBS, kept under magnetic stirring at 32.5 ± 0.5 °C and the entire cell was protected from ambient light. A volume of 500 μL was withdrawn from the receptor compartment, in 30 min intervals, and replaced by an equivalent volume of fresh PBS solution. The withdrawn samples were immediately analyzed by UV–visible spectrophotometry. The amount of intact GSNO diffused through the membrane was determined by monitoring the spectral changes at 336 nm (ε = 980 mol^−1^·L·cm^−1^), and assigned to the presence of intact GSNO [33,34,40]. The results were reported as the mean ± standard deviation (SD) of three independent experiments and expressed in terms of the GSNO percentage.

### 2.10. Mathematical Models

In order to investigate the mechanism of GSNO diffusion from the PL/CS hydrogel, the Higuchi mathematical model was applied to the kinetic curve obtained in item 2.9 (Equation (3)) [42]:

*Q*_t_ = *K*_H_*t*^0.5^, (3)

where *Q*_t_ is the cumulative amount of drug released at time, *t*, *K*_H_ is the release constant, and *t* is the time [33,43,44].

### 2.11. Cytotoxicity of CS/PL and GSNO-PL/CS Hydrogels

The cytotoxicity effects of CS/PL and GSNO-CS/PL hydrogels were evaluated towards Vero cells by MTT-3-(4,5-dimethylthiazol-2-yl)-2,5-diphenyltetrazolium bromide) assay [45,46]. Vero cells passage 173 (CCIAL-057) were seeded in 24-well plates. After 24 h, at a confluence of 80% and after forming a monolayer, the culture medium was removed and substituted by elution medium, prepared using cell culture medium and the hydrogels. The hydrogels were added to each well containing Vero cells at concentrations of 0.2, 1.2, 2.4, 6.0 and 9.5 µg·mL^−1^ of PL/CS hydrogel. These concentrations correspond to 0.2, 1.2, 2.4, 6.0 and 9.5 mmol·L^−1^ of GSNO, respectively (final concentrations. After adding each formulation to Vero cells, they were incubated for 24 h at 37 °C in a 5% CO_2_ atmosphere. The cells in the culture medium without any treatment were employed as the negative control, and a solution of phenol (0.25%) was used as a positive control (cytotoxic) [47,48,49]. After Vero cell incubation for 24 h with the PL/CS hydrogel and GSNO-PL/CS hydrogel, a volume of 100 µL of 10% MTT solution (5 mg·mL^−1^) in PBS was added to each of the wells, and the cells were incubated for an additional 4 h. After removing MTT solution, 50 μL of dimethyl sulfoxide was added to each well for 10 min, and the absorbance at 570 nm was measured with an automated spectrophotometric microtiter plate reader (SpectraMax M5, Molecular Devices, Sunnyvale, CA, USA). The experiment was performed in triplicate. The results were presented as the mean ± standard deviation (SD). The ANOVA test (one-way) was done, followed by the post hoc Tukey test for comparison between the two groups. Statistical significance was set at a *p*-value of ≤0.001.

### 2.12. Morphological Evaluation of Vero Cells after Treatment with the Hydrogels

The morphology of Vero cells after 24 h incubation with pure PL/CS or GSNO-PL/CS hydrogels (at final hydrogel concentration of 0.2–9.5 µg·mL^−1^) was observed with an inverted microscope (AxioVert A1, Zeiss, Jena, Germany) and phase contrast [49,50].

### 2.13. Antibacterial Activity of PL/CS and GSNO-PL/CS Hydrogels

The minimal inhibitory concentration (MIC) and minimal bactericidal concentration (MBC) of the PL/CS and GSNO-PL/CS hydrogels were carried out by microdilution assay, as described by the Clinical and Laboratory Standards Institute [51]. The bacterial strain evaluated was the gram-negative (standard CLSI) *Pseudomonas aeruginosa* ATCC 27853 (PAR). Initially, the bacteria were grown in Mueller–Hinton solid medium at 37 °C to obtain the isolated colonies. Subsequently, the colonies were solubilized in a saline solution 0.85% (*w*/*v*) and adjusted to the 0.5 index of the MacFarland scale (1.5 × 108 colony-forming units (cfu) per mL). This solution was diluted in Mueller–Hinton broth and distributed in a 96-well plate at a density of 106 cfu/well. Each well was treated with different concentrations of PL/CS and GSNO-PL/CS hydrogels of 0.3, 0.5, 1.0, 2.1, 3.15 and 4.2 µg·mL^−1^, which correspond to NO final concentrations of 0.5, 1.0, 2.0, 4.0, 6.0 and 8.0 mmol·L^−1^, respectively. The plates were incubated for 18 h and the bacterial growth was measured after this period. The MBC tests were performed after the MIC tests. After 24 h of incubation, drops of bacterial broth from the cavity with no visual growth were added to a Petri dish with the Mueller–Hinton solid medium, and bacterial growth was observed after 48 h. These tests were carried out in triplicate.

## 3. Results and Discussion

Biocompatible hydrogels might simulate living tissues more than any other class of synthetic biomaterial, due to their similarities [31,52,53]. They are easy to apply into several shapes and surfaces because of their softness. Thus, they are predominantly used in biomedical and topical applications [31]. PL hydrogels are versatile materials since it is possible to incorporate either hydrophilic [22,40,49,54] or hydrophobic drugs into PL hydrogels [55].The CS-based biomaterials have gained a lot of attention due to their biocompatibility, biodegradability and hemostatic properties for drug delivery and tissue engineering [38,56,57,58]. The interaction of positive protonated CS amino groups with the negatively charged mucin layer and tight junctions can facilitate the transport of hydrophilic macromolecules through the mucosal barrier [59]. This section describes and discusses the characterization of PL/CS as biocompatible hydrogel for topical applications in NO-therapy.

### 3.1. Characterization of PL/CS and GSH-PL/CS Hydrogels

**Figure 2:**
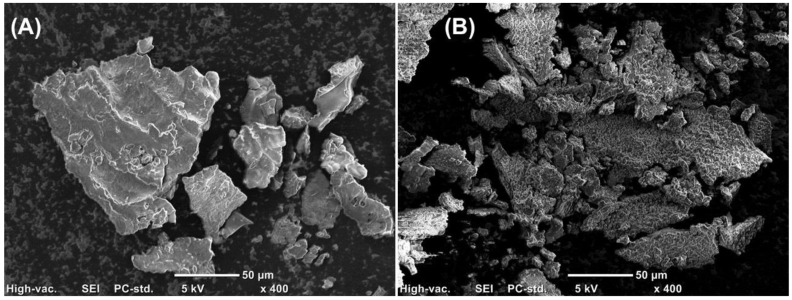
Scanning electron microscopy (SEM) micrographs of lyophilized (**A**) PL/CS hydrogel and (**B**) GSH-PL/CS hydrogel, using a 400× of magnification. The scale bar represents 50 µm.

In this study, GSH is the precursor of the NO donor molecule, GSNO [60]. Due to the intrinsic thermal and photochemical instability of GSNO [7,22,40], the characterization of morphological, rheological and thermal properties of the hydrogel was performed with the GSH-PL/CS hydrogel. The morphology of PL/CS and GSH-PL/CS hydrogels was observed by SEM analyses of lyophilized formulations [24,37]. Figure 2A exhibits a representative image of the PL/CS hydrogel. A smooth surface with defined edges and amorphous structures can be observed. Figure 2B exhibits a representative image of the GSH-PL/CS hydrogel. The GSH incorporation into the hydrogel leads to a more irregular surface. It should be noted that dynamic surfaces, as observed in Figure 2B, might increase cell adhesion, migration and detachment, which is desirable for tissue engineering applications [61]. The surface of hydrogel is the initial and primary site of interaction with surrounding cells and tissues. In biomedical applications, including in tissue engineering, hydrogels with roughness surfaces are considered suitable for cell attachment, cell proliferation, tissue growth, passage of nutrients, drug loading and delivery. In this sense, the surface morphology of the hydrogel (presence of pores and roughness) can affect the biomaterial biocompatibility through modifications in cell proliferations and attachment to a solid substrate. As shown in Figure 2, the GSH-PL/CS hydrogel has a roughness surface, while PL/CS hydrogel has a smooth surface. Roughness surface morphology might increase the cell attachment and the cell proliferation, which are desirable for tissue engineering applications [61]. The hydrogels studied here have similar morphologies to those described by Leyva-Gomes et al., who characterized PL and PL/CS hydrogels prepared by gamma irradiation [37].

Due to the presence of PL into the hydrogels, PL/CS and GSH-PL/CS hydrogels became thermoresponsive hydrogels [62]. At low temperatures (0–5 °C), PL/CS and GSH-PL/CS hydrogels had viscous behavior, whereas at higher temperatures (20–40 °C) the hydrogel had a solid-like behavior [62]. The gelling temperature (*T*_gel_) indicated the temperature required to change the rheological property of the material, and is understood as when elastic modulus (*G*′) becomes higher than loss modulus (*G*″) during a temperature ramp [63]. This feature can be related to the presence of PL into the hydrogel matrix, and has attracted significant interest involving drug release applications and tissue engineering [64].

*T*_gel_ values of PL/CS and GSH-PL/CS hydrogels were found to be 28.72 ± 0.98 °C and 29.95 ± 0.85 °C, respectively (Table 1). The GSH incorporation on the PL/CS hydrogel slightly increased *T*_gel_ by 1 °C. Kkatheb et al. showed a similar result upon the addition of laxacin into PL hydrogel, where their results showed an increase of *T*_gel_ approximately of 1 °C [65].

**Figure 3:**
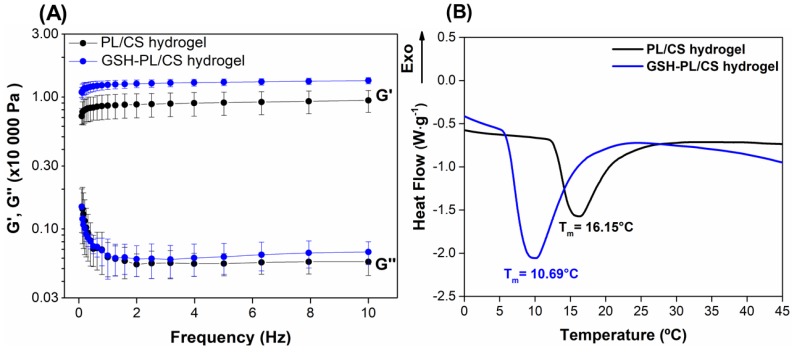
(**A**) Elastic (*G*′) and loss (*G*″) moduli of PL/CS hydrogel (black line) and GSH-PL/CS hydrogel (blue line) during an oscillation sweep frequency test at 32.5 °C; (**B**) Differential scanning calorimetric (DSC) thermographs of PL/CS hydrogel (black line) and GSH-PL/CS hydrogel (blue line).

Oscillation sweep frequency tests were performed on PL/CS and GSH-PL/CS hydrogels at 32.5 °C (the skin temperature) to evaluate the effect of GSH incorporation under the linear viscoelastic region [64]. Figure 3A shows that the *G*′ and *G*″ moduli did not significantly change in the applied frequency range (0.1–10 Hz). In addition, there was a linear region with a similar profile for PL/CS and GSH-PL/CS hydrogels for all tested frequencies (Figure 3A). Thus, the GSH incorporation into PL/CS hydrogel did not significantly change the hydrogel mechanical and gelling properties.

DSC is an important technique to evaluate the temperature that induces thermal transitions in solutions of thermoresponsive polymers [65]. Figure 3B exhibits the thermographs of PL/CS and GSH-PL/CS hydrogels, with the aim to investigate thermal transition changes after GSH incorporation. The PL/CS hydrogel showed an endotherm peak with an onset temperature (*T*_onset_) at 12.71 °C, a peak at 16.15 °C and an end set temperature (*T*_endset_) of 19.48 °C (Table 1). The endotherm peak can be related to the critical micellization temperature (CMT) process. In addition, *T*_onset_ is mainly related to the beginning of micelle formation, whereas *T*_endset_ shows the completion of the micelle formation process [65]. The incorporation of GSH into the PL/CS hydrogel decreased the *T*_onset_ by 6.14 °C, CMC by 5.46 °C and *T*_endset_ by 4.73 °C (Table 1 and Figure 3B). Thus, the GSH incorporation decreased the CMT by 40%. The micelle formation process is related to the dehydration of PPO block units in PL structure. The incorporation of GSH (hydrophilic molecule) is expected to form into a hydrophilic layer of the micelle, so the presence of GSH caused the dehydration of PPO block and, consequently, decreased the CMT value. The same trend was observed by Nie et al. by adding paclitaxel into PL hydrogel [22,42].

Enthalpy variation (∆H) is the area under the endothermic peak related to CMC. The ∆H values of PL/CS hydrogel and GSH-PL/CS hydrogel were found to be 4.013 and 4.237 J·g^−1^, respectively (Table 1). All formulations showed a positive ∆*H*. This result is related to the dehydration of the PPO block, which is an endothermic process. Thus, GSH incorporation into PL/CS hydrogel increased the ∆*H* value by 10%. Akkari et al. have shown a similar behavior by adding budesonide to PL hydrogels [66].

Taking together, the morphological, rheological and thermal properties of the GSH-PL/CS hydrogel were found to be suitable for drug delivery, since thermoresponsive behavior and desirable stability against oscillation were observed. These combined features make this hydrogel a potential candidate for topical applications of active molecules, such as NO/NO donors [31].

### 3.2. NO Release from GSNO-PL/CS Hydrogel

GSH is the precursor molecule of the NO donor, GSNO. In this work, GSH was nitrosated by reacting with an equimolar amount of NaNO_2_, in acid medium, forming the GSNO (Equation (4)). In this case, the nitrosating agent was nitrous acid (HNO_2_), which is formed by the dissolution of NO_2_^−^ in aqueous acidified solution. Once synthesized, GSNO was incorporated into PL/CS hydrogel:

GSH + HNO_2_ → GSNO + H_2_O. (4)

In this work, the kinetics of NO release from GSNO (either dissolved in aqueous solution or incorporated into GSNO-PL/CS hydrogel) was monitored for 24 h, at different temperatures. GSNO undergoes to a spontaneous decomposition releasing free NO and yielding oxidized glutathione (GSSG) (Equation (5)) [1,9,22,33]:

2 GSNO → 2 NO + GSSG.(5)

This reaction occurs through the homolytic cleavage of the S–N bond, which can be catalyzed by the presence of metal ions (e.g., copper), light and heat [1,9,22,33].

**Figure 4:**
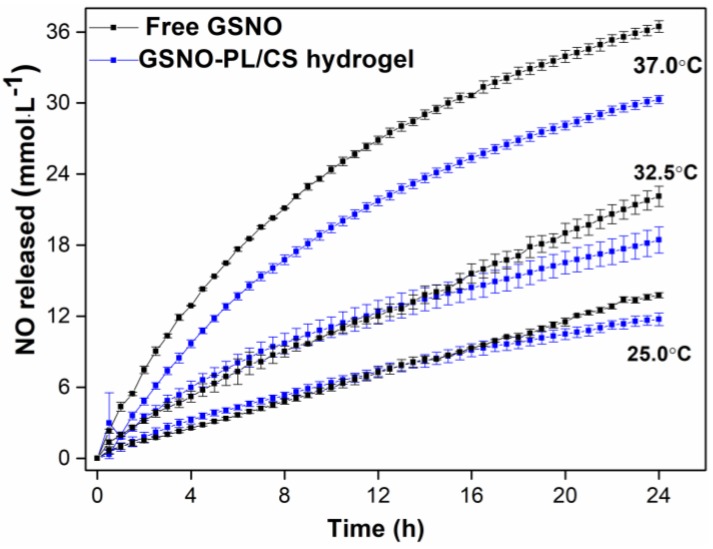
Kinetics of NO release from GSNO (initial concentration of 50 mmol·L^−1^) in aqueous solution (black lines) and incorporated into PL/CS hydrogel (blue lines), at 25, 32.5 and 37 °C. The results are reported as the mean ± standard deviation (SD) of three independent experiments.

Figure 4 shows the NO release profile from free GSNO and from GSNO-PL/CS hydrogel at different temperatures (25, 32.5 and 37 °C) for 24 h. As the stability of GSNO is known to be dependent on the temperature [39], the NO release profile was monitored at room, skin and physiological temperatures (25, 32.5 and 37 °C, respectively). Figure 4 shows that both GSNO in aqueous solution, and GSNO incorporated into the hydrogel matrix, were able to release NO for at least 24 h at the different tested temperatures. In addition, the increase of the temperature increased the rates of NO release from both aqueous GSNO and hydrogel containing GSNO. This result is in accordance with previous publications [7,9,22,30].

Furthermore, the incorporation of GSNO into PL/CS hydrogel decreased the rates of NO release, compared to GSNO dissolved in aqueous solution (Figure 4), as previously reported, when complexed to other materials [9,20,54]. This decrease was assigned to the cage effect promoted by the higher viscosity of the hydrogel matrix, in comparison to the aqueous environment. The superior viscosity of the hydrogel matrix favors the radical pair recombination of NO^•^ and GS^•^, restoring the GSNO molecule, and increasing the rates of NO release. In this sense, the viscosity of the medium can be used to modulate the rates of GSNO decomposition, and consequently the rates of NO release [31].

Figure 4 shows that the concentration of NO release from GSNO was in the millimolar range. At this range, NO is expected to have therapeutic effects [60], such as antibacterial activity. After 24 h at 37 °C, the total concentration of NO release from aqueous GSNO was ca. 36 mmol·L^−1^, while the concentration of NO release from the hydrogel was 30 mmol·L^−1^ (Figure 4). The incorporation of GSNO into the PL/CS hydrogel decreased the amount of total NO released by 20%, at the same period of time.

It should be noted that for therapeutic purposes, a sustained and localized release of NO is desirable. Figure 4 shows that the incorporation of GSNO into PL/CS hydrogel promotes a sustained NO release, in concentrations suitable for biomedical applications, for at least 24 h, at physiological and skin temperatures. In addition, the hydrogel allows an increase in residence time of the NO donor directly on the target site of application, making this formation a good candidate for a topical NO delivery system [1,7,67,68,69].

### 3.3. GSNO Release from GSNO-PL/CS Hydrogel

**Figure 5:**
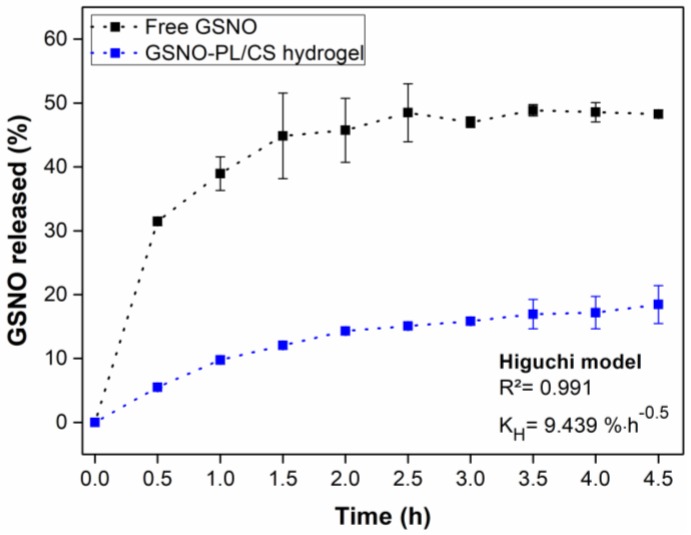
In vitro release profile of free GSNO (black line) and GSNO-PL/CS hydrogel (blue line), using a Franz diffusion cell system at 32.5 °C (initial GSNO concentration of 50 mmol·L^−1^). The results are reported as the mean ± standard deviation (SD) of three independent experiments.

The vertical Franz diffusion cell was used to evaluate the diffusion of intact GSNO through a membrane versus GSNO dissolved in aqueous solution and GSNO-PL/CS hydrogel, at 32.5 °C (skin temperature). Figure 5 shows that in both cases, a two-phase release profile of GSNO was observed, which included: (1) an initial burst of GSNO diffusion (within the first 1.5 h of monitoring); and (2) the establishment of a plateau, which corresponded to a steady-state of GSNO diffusion release [20,70]. This diffusion profile is similar to other reports published by the authors [30,32,33]. Additionally, Wu et al. showed a similar GSNO release profile from free GSNO and GSNO encapsulated into polymer nanocomposite particles [71].

The diffusion of GSNO from aqueous GSNO reached a value of 50% of the total GSNO content after 4.5 h of monitoring, whereas in the case of GSNO-PL/CS hydrogel the amount of GSNO that diffused was 20%, at the same time frame (Figure 5). Indeed, the incorporation of GSNO into the PL/CS hydrogel decreased the amount that GSNO diffused by 60%. This result is in accordance with Figure 4 (i.e., kinetics of NO release from aqueous and GSNO-containing hydrogel). The superior viscosity of the hydrogel matrix, related to the aqueous solution, promotes sustained and controlled GSNO diffusion from the hydrogel matrix, making this formulation suitable for topical applications.

The Higuchi mathematical model was fit to the data presented in Figure 5 with a correlation coefficient (*R*^2^) of 0.991. This indicates a Fickian diffusion of GSNO from GSNO-PL/CS hydrogel with a constant (*K*_H_) of 9.439% h^−1^. Similar studies involving *S*-nitrosothiols showed a release mechanism mainly limited by the Fickian diffusion. For example, Oliveira et al. showed the Fickian release of NO from chitosan nanoparticles for agriculture applications [39], and Pelegrino et al. showed a Fickian diffusion of GSNO from pure PL hydrogels or from hydrogel containing chitosan nanoparticles [32]. Therefore, GSNO incorporation into the PL/CS hydrogel significantly decreased the rates GSNO diffusion from the hydrogel matrix, allowing a sustained release of the NO donor directly onto the desired site of application.

### 3.4. Cytotoxicity of PL/CS and GSNO-PL/CS Hydrogels

The biocompatibility of the GSNO-PL/CS hydrogel was accessed as an in vitro cytotoxicity test using Vero cells, a lineage mentioned at the ISO 10993-5 [47] standard for these tests. These adherent cells show fibroblastic morphology and express cytotoxicity results in contact with different biomaterials and solutions [72,73,74].

Cytotoxicity assays were performed by varying concentrations of the PL/CS and GSNO-PL/CS hydrogels. Namely, 0.2, 1.2, 2.4, 6.0 and 9.5 µg·mL^−1^ of PL/CS hydrogel were used, and these concentrations correspond to 0.2, 1.2, 2.4, 6.0 and 9.5 mmol·L^−1^ of GSNO, respectively (in the case of GSNO-containing hydrogel). Cytotoxicity was assessed by MTT assay, using the Vero cell line, and by morphological cell characterization after cell incubation with the hydrogels for 24 h.

**Figure 6:**
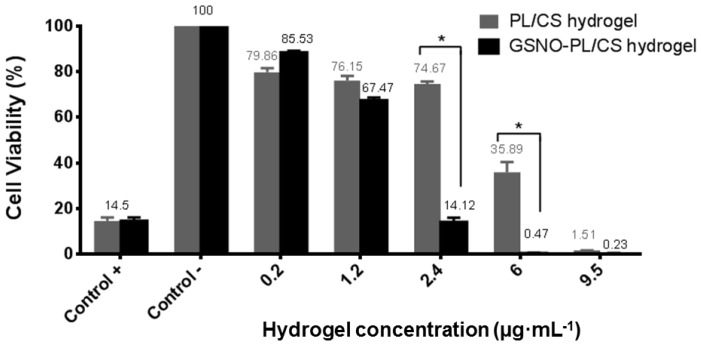
Vero cell viability after incubation with PL/CS and GSNO-PL/CS hydrogels (at final hydrogel concentrations of 0.2–9.5 µg·mL^−1^) for 24 h. The cells in the culture medium without any treatment were employed as the negative control, and a solution of phenol (0.25%) was used as a positive control.

Figure 6 shows that both hydrogels showed a concentration-related cytotoxicity. For the PL/CS hydrogel, concentrations under 2.4 µg·mL^−1^ were considered noncytotoxic. However, in the presence of GSNO-containing hydrogel, the cytotoxicity of the formulation towards Vero cells was observed at concentrations under 1.2 µg·mL^−1^. As expected, the hydrogels did not significantly decrease cell viability at lower concentrations. A decrease in cell viability was found for the higher tested concentrations.

**Figure 7:**
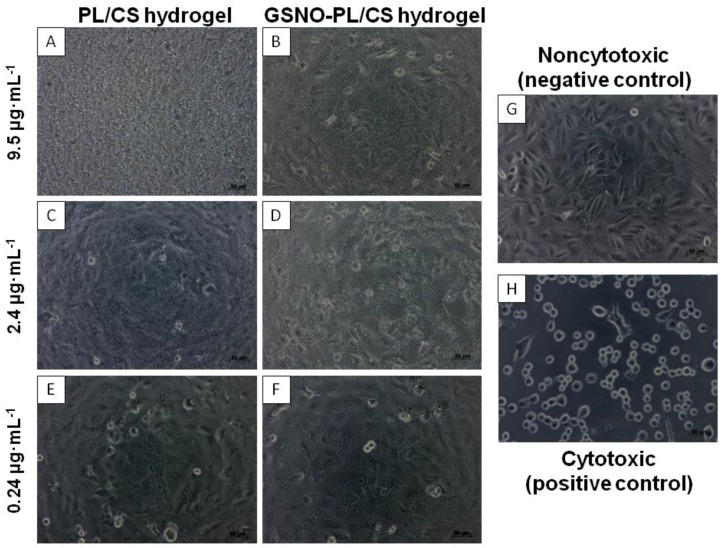
Morphological characterization of Vero cells incubated with PL/CS hydrogel (first column) and GSNO-PL/CS hydrogel (second column) at 9.5 µg·mL^−1^ ((**A**,**B**), respectively), 2.4 µg·mL^−1^ ((**C**,**D**), respectively) and 0.24 µg·mL^−1^ ((**E**,**F**), respectively) for 24 h. Control samples (third column) of non-cytotoxicity (**G**) and cytotoxicity (**H**).

The cytotoxic pattern is shown in Figure 7A–F. Noncytotoxic Vero cells grew in a confluent monolayer, with a fibroblastic-like polygonal morphology (Figure 7G). With the presence of a cytotoxic compound, namely 0.25% phenol, the cell showed a rounded and loose morphology, non-attached, and lifted from the culture plates, with signs of cell death (Figure 7H). These parameters were used to analyze the different tested compounds, PL/CS and GSNO-PL/CS hydrogel.

The predominance of the PL/CS-hydrogel can be observed in Figure 7A,B, which consequently covered and/or caused the death of Vero cells. With the decrease in the concentration of the tested components, the presence of the cells, and morphology similar to the non-cytotoxic control, were observed (Figure 7C–F).

At hydrogel concentrations higher than 1.2 μg·mL^−1^, the presence of rounded and debris cells can be observed, a typical characteristic of cytotoxicity (Figure 7B,D). These results demonstrated a concentration-dependent cytotoxicity of the GSNO-PL/CS hydrogel to the Vero cell line. These data are in accordance with a previous publication by the authors, which demonstrated that high NO levels are cytotoxic to different normal and cancerous cell lines [33,75]. These findings are also in agreement with several publications that demonstrated a concentration-dependence toxicity of different classes of NO donors towards health and tumor cell lines [33,60,76,77,78].

The proposed mechanisms of cytotoxicity caused by the NO donors involves the multifunctional transcription factor NF-κB, inhibition of both ribonucleotide reductase and ATP, or protein expression mediators [33,76,78]. Ferraz et al. studied the cytotoxicity mechanism of *S*-nitrosothiol-containing polymeric nanoparticle (NO-NP) against a melanoma cell line [75]. The NO-NP induced apoptosis against melanoma cells with the activation of effector caspase 3. Additionally, an increase of oxidative stress in the cells incubated with NO-NP was observed, which was related to an increase generation of a superoxide anion radical by mitochondria [75].

In contrast, low concentrations of both hydrogels were not toxic to Vero cells (Figure 6). The biocompatibility of systems composed of PL/CS hydrogels has been demonstrated through various applications [24,49,79]. The thermosensitive response of the PL/CS hydrogel has been applied as an injectable scaffold and as a cell delivery carrier matrix for tissue regeneration [36,79,80,81]. Olguín et al. reported advanced properties of this thermosensitive hydrogel for the delivery of cells in neural tissue engineering, and demonstrated a delicate relationship between physical properties and capabilities to promote cell proliferation and differentiation [79].

PL/CS hydrogels have also been applied as carrier systems for drug, gene and biomolecule delivery [24,82,83,84,85,86]. The common points of these studies was to combine and explore suitable features of each polymer (PL and CS) in a single formation, i.e., mucoadhesion and thermogelling. These were intended to prolong the residence time of the delivery systems, thus improving the efficacy of local or systemic drugs/biomolecules [87]. Radivojša et al. developed a novel approach based on thermoresponsive hydrogels with CS nanocomplexes for prolonged release of low molecular weight heparins (LMWH). These double-responsive mixed preparations seemed to be promising systems for subcutaneous LMWH delivery, requiring less frequent administration during long-term treatment. Additionally, formulations demonstrated no cytotoxicity in vitro for human keratinocyte cells [84]. The application of hydrogels as release systems for NO donor compounds has also been described in the literature. De Oliveira (2016) developed a topical hydrogel formulation comprising Pluronic F127 containing GSNO in concentrations ranging from 50 to 150 mmol·L^−1^ [39]. These formulations were shown to produce a significant dermal vasodilation in topical applications on the healthy skin of volunteers [39,40].

### 3.5. Antibacterial Activity of PL/CS and GSNO-PL/CS Hydrogels

The antimicrobial properties of PL/CS and GSNO-PL/CS hydrogels were investigated against gram-negative bacteria, *Pseudomonas aeruginosa* (PAR). PAR is an important human pathogen, responsible for a wide range of chronic and acute infections, such as diabetic foot infections, burn wounds and pneumonia in cystic fibrosis patients [88,89,90,91,92,93,94]. PAR is particular resistant bacteria against β-lactans antibiotics due to its intrinsic capability of expressing β-lactamases and efflux pumps [91].

Both MIC and MBC values for PL/CS hydrogel were found to be 2.1 µg·mL^−1^, whereas both MIC and MBC values for GSNO-PL/CS hydrogel were found to be 0.5 µg·mL^−1^ (this concentration corresponds to 1 mmol·L^−1^ of GSNO) (Table 2). The sole PL/CS hydrogel had a bacterial effect; however, the incorporation of GSNO into the hydrogel decreased both MIC and MBC values by four times. Thus, as expected, the PL/CS hydrogel demonstrated antibacterial activity (due to the presence of CS), and this activity was enhanced by GSNO incorporation into the hydrogel matrix. Interesting, by comparing Table 2 and Figure 6, the hydrogel concentration required to achieve an anti-bacterial effect was not found to be cytotoxic (as related to a cytotoxicity pattern in Vero cells). Therefore, there is a concentration range that the GSNO-containing PL/CS hydrogel that can be safely administered to the patient, without damaging mammalian cells, and with a promising antibacterial effect.

Similar to our results, Hetrick et al. showed a treatment composed of nitrosated proline as an NO donor (PROLI/NO) against PAR. The treatment exhibited a MIC value of 2.5 mg·mL^−1^ [95]. Seabra et al. demonstrated the antibacterial effects of NO-releasing polyester [10] and NO-releasing-AgNPs towards different gram positive and negative bacteria strains [2]. Barraud et al. studied the effect of NO donor, sodium nitroprusside (SNP), against PAR strains in planktonic and biofilm forms [96]. At concentration of 5 µmol·L^−1^ of SNP, a decrease of 50% in the CFU of planktonic PAR was observed. In addition, the authors observed that this effect was decreased when the NO treatment was combined with a phosphodiesterases inhibitor. Thus, the possible antibacterial mechanism of NO is related to phosphodiesterases, which contributes to a genetic network that modulates virulence and biofilm formation and dispersion [96].

NO is known to have a potent and wide range of antimicrobial effects against both gram-negative and gram-positive bacteria [60]. The main mechanism by which NO kills bacteria is understood to involve nitrosative stress, which evolves the production of reactive nitrogen/oxygen species. As the antibacterial effect of NO involves multiple pathways, bacteria have, thus far, not been able to develop resistance towards NO [60].

CS is also known to have antibacterial effects [82,88]. The main antibacterial effects caused by CS include: (1) cell penetration and interaction with DNA; (2) CS chelation of metal ions, which leads to the production of toxins, inhibition of enzymatic activity, and finally the cell cycle; and (3) electrostatic interactions between positively charged CS and negatively charged cell membrane [88].

Thus, a biomaterial that combines the antimicrobial activities of CS and NO might be considered a potent antimicrobial agent. In addition, the viscosity of the semi-solid hydrogel matrix allows its topical/dermatological applications, increasing the contact time and, therefore, the interaction of the formulation with application target sites. In this sense, the NO/NO donor can be directly released from the hydrogel matrix. This approach might find important applications in the treatment of skin and soft tissue infections, such as diabetic foot infections, otitis externa and burn wounds [94].

## 4. Conclusions

This work described the preparation, characterization, NO/GSNO release profile, biocompatibility and antibacterial effects of GSNO-containing PL/CS hydrogel. This hydrogel can be considered as a promising system for NO delivery due to its biocompatibility, easy preparation, ability to incorporate NO donors, ability to promote a sustained NO and GSNO release and antibacterial effect. Their main features of the hydrogel include smooth morphology, thermoresponsive behavior and good mechanical stability. In addition, this system is free of crosslink agents, increasing the biocompatibility of the hydrogel, due to the absence of possible toxic sub-products. The GSNO-PL/CS hydrogel was found to release therapeutic amounts of NO in a sustainable manner. GSNO-containing PL/CS hydrogel was not toxic towards the Vero cell at concentrations lower than 13.23 μg·mL^−1^, which increases the safety aspect of this material. GSNO-containing PL/CS hydrogel demonstrated antibacterial effects against *Pseudomonas aeruginosa*, with MIC and MBC values of 0.5 µg·mL^−1^ (this concentration corresponds to 1 mmol·L^−1^ of GSNO). Importantly, the antibacterial effect of the NO-releasing hydrogel was observed at concentrations that were not toxic to the Vero cell.

## Figures and Tables

**Table 1:** *T*_onset_ (°C), critical micellar temperature (CMT) (°C), *T*_endset_ (°C) and ∆*H* (J·g^−1^) acquired using DSC analyses and *T*_sol/gel_ (°C) acquired using rheology analyses of PL/CS and GSH-PL/CS hydrogels.

Material	*T*_onset_ (°C)	CMT (°C)	*T*_endset_ (°C)	∆*H* (J·g^−1^)	*T*_gel_ (°C)
PL/CS	12.71	16.15	19.48	4.013	28.72 ± 0.98
GSH-PL/CS	6.57	10.69	14.75	4.237	29.95 ± 0.85

**Table 2:** Values of minimal inhibitory concentration (MIC) and minimal bactericidal concentration (MBC) for PL/CS and GSNO-PL/CS hydrogels against *Pseudomonas aeruginosa* (PAR) ATCC 27853, as model microorganism.

Material	*Pseudomonas aeruginosa* (PAR) ATCC 27853	
	MIC (µg·mL^−1^)	MBC (µg·mL^−1^)
PL/CS hydrogel	2.1	2.1
GSNO-PL/CS hydrogel	0.5	0.5

## References

[1] Seabra A.B., Justo G.Z., Haddad P.S. (2015). State of the art, challenges and perspectives in the design of nitric oxide-releasing polymeric nanomaterials for biomedical applications. Biotechnol. Adv..

[2] Seabra A.B., Elgueta N.M., Lima B.A., Pelegrino M.T., Brocchi M., Rubilar O., Durán N. (2017). Antibacterial activity of nitric oxide releasing silver nanoparticles. J. Phys. Conf. Ser..

[3] Iganarro L., Freeman B. (2017). Nitric Oxide Biology and Pathobiology.

[4] Carpenter A.W., Schoenfisch M.H. (2012). Nitric oxide release Part II. Therapeutic applications. Chem. Soc. Rev..

[5] Seabra A.B., da Silva R., de Souza G.F.P., de Oliveira M.G. (2008). Antithrombogenic polynitrosated polyester/poly(methyl methacrylate) blend for the coating of blood-contacting surfaces. Artif. Organs.

[6] Ignarro L.J., Buga G.M., Wood K.S., Byrns R.E., Chaudhuri G. (1987). Endothelium-derived relaxing factor produced and released from artery and vein is nitric oxide. Proc. Natl. Acad. Sci. USA.

[7] Seabra A.B., Fitzpatrick A., Paul J., de Oliveira M.G., Weller R. (2004). Topically applied S-nitrosothiol-containing hydrogels as experimental and pharmacological nitric oxide donors in human skin. Br. J. Dermatol..

[8] Fukuto J.M., Carrington S.J., Tantillo D.J., Harrison J.G., Ignarro L.J., Freeman B.A., Chen A., Wink D.A. (2012). Small molecule signaling agents: The integrated chemistry and biochemistry of nitrogen oxides, oxides of carbon, dioxygen, hydrogen sulfide, and their derived species. Chem. Res. Toxicol..

[9] Brisbois E.J., Handa H., Major T.C., Bartlett R.H., Meyerhoff M.E. (2013). Long-term nitric oxide release and elevated temperature stability with *S*-nitroso-*N*-acetylpenicillamine (SNAP)-doped Elast-eon E2As polymer. Biomaterials.

[10] Seabra A.B., Martins D., Simões M.M.S.G., da Silva R., Brocchi M., de Oliveira M.G. (2010). Antibacterial nitric oxide-releasing polyester for the coating of blood-contacting artificial materials. Artif. Organs.

[11] Wo Y., Brisbois E.J., Bartlett R.H., Meyerhoff M.E. (2016). Recent advances in thromboresistant and antimicrobial polymers for biomedical applications: Just say yes to nitric oxide (NO). Biomater. Sci..

[12] Frank S., Kämpfer H., Wetzler C., Pfeilschifter J. (2002). Nitric oxide drives skin repair: Novel functions of an established mediator. Kidney Int..

[13] Georgii J.L., Amadeu T.P., Seabra A.B., de Oliveira M.G., Monte-Alto-Costa A. (2011). Topical *S*-nitrosoglutathione-releasing hydrogel improves healing of rat ischaemic wounds. J. Tissue Eng. Regen. Med..

[14] Huang B., Liu M., Zhou C. (2017). Chitosan composite hydrogels reinforced with natural clay nanotubes. Carbohydr. Polym..

[15] Li Y., Wang X., Wei Y., Tao L. (2017). Chitosan-based self-healing hydrogel for bioapplications. Chin. Chem. Lett..

[16] Vercelino R., Cunha T.M., Ferreira E.S., Cunha F.Q., Ferreira S.H., de Oliveira M.G. (2013). Skin vasodilation and analgesic effect of a topical nitric oxide-releasing hydrogel. J. Mater. Sci. Mater. Med..

[17] Kellogg D.L., Liu Y., Kosiba I.F., O’Donnell D. (1999). Role of nitric oxide in the vascular effects of local warming of the skin in humans. J. Appl. Physiol..

[18] Miclescu A., Gordh T. (2009). Nitric oxide and pain: “Something old, something new”. Acta Anaesthesiol. Scand..

[19] Laftah W.A., Hashim S., Ibrahim A.N. (2011). Polymer hydrogels: A review. Polym. Plast. Technol. Eng..

[20] Malli S., Bories C., Pradines B., Loiseau P.M., Ponchel G., Bouchemal K. (2017). In situ forming pluronic F127/chitosan hydrogel limits metronidazole transmucosal absorption. Eur. J. Pharm. Biopharm..

[21] Akash M.S.H., Rehman K. (2015). Recent progress in biomedical applications of pluronic (PF127): Pharmaceutical perspectives. J. Control. Release.

[22] Shishido S.M., Seabra A.B., Loh W., de Oliveira M.G. (2003). Thermal and photochemical nitric oxide release from *S*-nitrosothiols incorporated in Pluronic F127 gel: Potential uses for local and controlled nitric oxide release. Biomaterials.

[23] De Araujo D.R., Oshiro A., da Silva D.C., Akkari A.C., de Mello J.C., Rodrigues T., Durán N., Guterres S.S., Alves O.L. (2013). Poloxamers as Drug-Delivery Systems:Physicochemical, Pharmaceutical, and Toxicological Aspects. Nanotoxicology: Materials, Methodologies, and Assessments.

[24] Yu S., Zhang X., Tan G., Tian L., Liu D., Liu Y., Yang X., Pan W. (2017). A novel pH-induced thermosensitive hydrogel composed of carboxymethyl chitosan and poloxamer cross-linked by glutaraldehyde for ophthalmic drug delivery. Carbohydr. Polym..

[25] Zou P., Yang X., Wang J., Li Y., Yu H., Zhang Y., Liu G. (2016). Advances in characterisation and biological activities of chitosan and chitosan oligosaccharides. Food Chem..

[26] Divya K., Vijayan S., George T.K., Jisha M.S. (2017). Antimicrobial properties of chitosan nanoparticles: Mode of action and factors affecting activity. Fibers Polym..

[27] Seabra A.B., Pelegrino M.T. (2017). Chitosan-based nanomaterials for skin regeneration. AIMS Med. Sci..

[28] Fernandez J.G., Ingber D.E. (2013). Bioinspired chitinous material solutions for environmental sustainability and medicine. Adv. Funct. Mater..

[29] Schmolka I.K. (1972). Artificial skin: Preparation and properties of Pluronic F-127 gels for treatment of burns. J. Biomed. Mater. Res..

[30] Pelegrino M.T., de Araújo D.R., Seabra A.B. (2018). *S*-nitrosoglutathione-containing chitosan nanoparticles dispersed in Pluronic F-127 hydrogel: Potential uses in topical applications. J. Drug Deliv. Sci. Technol..

[31] Champeau M., Seabra A.B., de Oliveira M.G., Seabra A.B. (2017). Hydrogels for topical nitric oxide delivery. Nitric oxide Donors: Novel Biomedical Applications and Perspectives.

[32] Pelegrino M.T., Weller R.B., Chen X., Bernardes J.S., Seabra A.B. (2017). Chitosan nanoparticles for nitric oxide delivery in human skin. Med. Chem. Commun..

[33] Pelegrino M.T., Silva L.C., Watashi C.M., Haddad P.S., Rodrigues T., Seabra A.B. (2017). Nitric oxide-releasing nanoparticles: Synthesis, characterization, and cytotoxicity to tumorigenic cells. J. Nanopart. Res..

[34] Silveira N.M., Frungillo L., Marcos F.C.C., Pelegrino M.T., Miranda M.T., Seabra A.B., Salgado I., Machado E.C., Ribeiro R.V. (2016). Exogenous nitric oxide improves sugarcane growth and photosynthesis under water deficit. Planta.

[35] Chang F.C., Tsao C.T., Lin A., Zhang M., Levengood S.L., Zhang M. (2016). PEG-Chitosan hydrogel with tunable stiffness for study of drug response of breast cancer cells. Polymers.

[36] Yap L.S., Yang M.C. (2016). Evaluation of hydrogel composing of Pluronic F127 and carboxymethyl hexanoyl chitosan as injectable scaffold for tissue engineering applications. Coll. Surf. B.

[37] Leyva-Gómez G., Santillan-Reyes E., Lima E., Madrid-Martínez A., Krötzsch E., Quintanar-Guerrero D., Garciadiego-Cázares D., Martínez-Jiménez A., Hernández Morales M., Ortega-Peña S. (2017). A novel hydrogel of poloxamer 407 and chitosan obtained by gamma irradiation exhibits physicochemical properties for wound management. Mater. Sci. Eng. C.

[38] Wu T., Huang J., Jiang Y., Hu Y., Ye X., Liu D., Chen J. (2018). Formation of hydrogels based on chitosan/alginate for the delivery of lysozyme and their antibacterial activity. Food Chem..

[39] De Oliveira M.G. (2016). S-Nitrosothiols as Platforms for Topical Nitric Oxide Delivery. Basic Clin. Pharmacol. Toxicol..

[40] Seabra A.B., de Oliveira M.G. (2004). Poly(vinyl alcohol) and poly(vinyl pyrrolidone) blended films for local nitric oxide release. Biomaterials.

[41] Franz T.J. (1975). Percutaneous absorption on the relevance of in vitro data. J. Investig. Dermatol..

[42] Nie S., Hsiao W.L.W., Pan W., Yang Z. (2011). Thermoreversible pluronic F127-based hydrogel containing liposomes for the controlled delivery of paclitaxel: In vitro drug release, cell cytotoxicity, and uptake studies. Int. J. Nanomed..

[43] Costa P., Lobo J.M. (2001). Modeling and comparison of dissolution profiles. Eur. J. Pharm. Sci..

[44] Oshiro A., Silva D.C., Mello J.C., De Moraes V.W.R., Cavalcanti L.P., Franco M.K.K.D., Alkschbirs M.I., Fraceto L.F., Yokaichiya F., Rodrigues T. (2014). Pluronics F-127/L-81 binary hydrogels as drug-delivery systems: In fl uence of physicochemical aspects on release kinetics and cytotoxicity. Langmuir.

[45] Mosmann T. (1983). Rapid colorimetric assay for cellular growth and survival: Application to proliferation and cytotoxicity assays. J. Immunol. Meth..

[46] Kirkpatrick C.J., Bittinger F., Wagner M., Köhler H., van Kooten T.G., Klein C.L., Otto M. (1998). Current trends in biocompatibility testing. Proc. Inst. Mech. Eng. H.

[47] International Standards Organization (2009). ISO 10993-5. Biological Evaluation of Medical Devices—Part 5: Tests for In Vitro Cytotoxicity.

[48] Rogero S.O., Lugão A.B., Íkeda T.I., Cruz A.S. (2003). Teste in vitro de citotoxicidade: Estudo comparativo entre duas metodologias. Mater. Res..

[49] Nascimento M.H.M., Ferreira M., Malmonge S.M., Lombello C.B. (2017). Evaluation of cell interaction with polymeric biomaterials based on hyaluronic acid and chitosan. J. Mater. Sci. Mater. Med..

[50] Lombello C.B., Malmonge S.M., Wada M.L.F. (2000). PolyHEMA and polyHEMA-poly(MMA-*co*-AA) as substrates for culturing cells. J. Mater. Sci. Mater. Med..

[51] CLSI (2015). Performance Standards for Antimicrobial Susceptibility Testing. Twenty-Fifth Informational Supplement CLSI Document M100-S25.

[52] Caló E., Khutoryanskiy V.V. (2015). Biomedical applications of hydrogels: A review of patents and commercial products. Eur. Polym. J..

[53] Domínguez-Delgado C.L., Fuentes-Prado E., Escobar-Chávez J.J., Vidal-Romero G., Rodríguez-Crus I.M., Díaz-Torres R. (2016). Chitosan and Pluronic® F-127: Pharmaceutical applications. Encyclopedia of Biomedical Polymers and Polymeric Biomaterials.

[54] Schanuel F.S., Santos K.S.R., Monte-Alto-Costa A., de Oliveira M.G. (2015). Combined nitric oxide-releasing poly(vinyl alcohol) film/F127 hydrogel for accelerating wound healing. Coll. Surf. B.

[55] Pellosi D.S., Moret F., Fraix A., Marino N., Maiolino S., Gaio E., Hioka K., Reddi E., Sortino S., Quaglia F. (2016). Pluronic® P123/F127 mixed micelles delivering sorafenib and its combination with verteporfin in cancer cells. Int. J. Nanomed..

[56] Li P., Zhao J., Chen Y., Cheng B., Yu Z., Zhao Y., Yana X., Tonga Z., Jina S. (2017). Preparation and characterization of chitosan physical hydrogels with enhanced mechanical and antibacterial properties. Carbohydr. Polym..

[57] Tan H., Chu C.R., Payne K., Marra K.G. (2009). Injectable in situ forming biodegradable chitosan-hyaluronic acid based hydrogels for cartilage tissue engineering. Biomaterials.

[58] Bernkop-Schnurch A., Dunnhaupt S. (2012). Chitosan-based drug delivery systems. Eur. J. Biopharm..

[59] Bowman K., Leong K.W. (2006). Chitosan nanoparticles for oral drug and gene delivery. Int. J. Nanomed..

[60] Seabra A.B. (2017). Nitric Oxide Donors: Novel Biomedical Applications and Perspectives.

[61] Uto K., Tsui J.H., Deforest C.A., Kima D. (2017). Dynamically tunable cell culture platforms for tissue engineering and mechanobiology. Prog. Polym. Sci..

[62] Klouda L. (2015). Thermoresponsive hydrogels in biomedical applications A seven-year update. Eur. J. Pharm. Biopharm..

[63] Olaru A.M., Marin L., Morariu S., Pricope G., Pinteala M., Tartau-Mititelu L. (2018). Biocompatible chitosan based hydrogels for potential application in local tumour therapy. Carbohydr. Polym..

[64] Dou Q., Karim A.A., Loh X.J. (2016). Modification of thermal and mechanical properties of PEG-PPG-PEG copolymer (F127) with MA-POSS. Polymers.

[65] Al Khateb K., Ozhmukhametova E.K., Mussin M.N., Seilkhanov S.K., Rakhypbekov T.K., Lau W.M., Khutoryanskiy V.V. (2016). In situ gelling systems based on Pluronic F127/Pluronic F68 formulations for ocular drug delivery. Int. J. Pharm..

[66] Akkari A.C.S., Campos E.V.R., Keppler A.F., Fraceto L.F., de Paula E., Tófoli G.R., de Araujo D.R. (2016). Budesonide-hydroxypropyl-cyclodextrin inclusion complex inbinary poloxamer 407/403 system for ulcerative colitis treatment: Aphysico-chemical study from micelles to hydrogels. Coll. Surf. B Biointerfaces.

[67] Marcilli R.H.M., de Oliveira M.G. (2014). Nitric oxide-releasing poly(vinyl alcohol) film for increasing dermal vasodilation. Coll. Surf. B.

[68] Taladriz-Blanco P., Pastoriza-Santos V., Pérez-Juste J., Hervés P. (2013). Controllable nitric oxide release in the presence of gold nanoparticles. Langmuir.

[69] Zhou X., Wang H., Zhang J., Li X., Wu Y., Wei Y., Ji S., Kong D., Zhao Q. (2017). Functional poly(ε-caprolactone)/chitosan dressings with nitric oxide-releasing property improve wound healing. Acta Biomater..

[70] Romić M.D., Klarić M.Š., Lovrić J., Pepić I., Cetina-Čižmek B., Filipović-Grčić J., Hafner A. (2016). Melatonin-loaded chitosan/Pluronic® F127 microspheres as in situ forming hydrogel: An innovative antimicrobial wound dressing. Eur. J. Pharm. Biopharm..

[71] Wu W., Gaucher C., Fries I., Hu X., Maincent P., Sapin-Mineta A. (2015). Polymer nanocomposite particles of *S*-nitrosoglutathione: A suitable formulation for protection and sustained oral delivery. Int. J. Pharm..

[72] Adams R.L.P. (1990). Cell Culture for Biochemists.

[73] Ammerman N.C., Beier-Sexton M., Azad A.F. (2008). Growth and maintenance of Vero cell lines. Curr. Protoc. Microbiol..

[74] Fernández-Freire P., Labrador V., Pérez Martín J.M., Hazen M.J. (2005). Cytotoxic effects in mammalian Vero cells exposed to pentachlorophenol. Toxicology.

[75] Ferraz L.S., Watashi C.M., Colturato-Kido C., Pelegrino M.T., Paredes-Gamero E.J., Weller R.B., Seabra A.B., Rodrigues T. (2018). Antitumor potential of *S*-nitrosothiol-containing polymeric nanoparticles against melanoma. Mol. Pharm..

[76] Sanina N., Shmatko N., Stupina T., Balakina A., Terent’ev A. (2017). NO-donor iron nitrosyl complex with *n*-ethylthiourea ligand exhibits selective toxicity to glioma A172 cells. Molecules.

[77] Lau H.K.F. (2003). Cytotoxicity of nitric oxide donors in smooth muscle cells is dependent on phenotype, and mainly due to apoptosis. Atherosclerosis.

[78] Huerta S., Chilka S., Bonavida B. (2008). Nitric oxide donors: Novel cancer therapeutics (review). Int. J. Oncol..

[79] Olguín Y., Campos C., Catalán J., Velásquez L., Osorio F., Montenegro I., Madrid A., Acevedo C. (2017). Effects of Liposomes Contained in Thermosensitive Hydrogels as Biomaterials Useful in Neural Tissue Engineering. Materials.

[80] Park K.M., Lee S.Y., Joung Y.K., Na J.S., Lee M.C., Park K.D. (2009). Thermosensitive chitosan-Pluronic hydrogel as an injectable cell delivery carrier for cartilage regeneration. Acta Biomater..

[81] Cellesi F. (2012). Thermoresponsive hydrogels for cellular delivery. Ther. Deliv..

[82] Choi J.S., Yoo H.S. (2010). Pluronic/chitosan hydrogels containing epidermal growth factor with wound-adhesive and photo-crosslinkable properties. J. Biomed. Mater. Res. A.

[83] Fu Y., Du L., Wang Q., Liao W., Jin Y., Dong A., Chen C., Li Z. (2012). In vitro sustained release of recombinant human bone morphogenetic protein-2 microspheres embedded in thermosensitive hydrogels. Pharmazie.

[84] Radivojša M.M., Grabnar I., Gosenca M., Grabnar P.A. (2015). Prolonged subcutaneous delivery of low molecular weight heparin based on thermoresponsive hydrogels with chitosan nanocomplexes: Design, in vitro evaluation, and cytotoxicity studies. Int. J. Pharm..

[85] Zhu X., Zhang Y., Huang H., Zhang H., Hou L., Zhang Z. (2016). Functionalized graphene oxide-based thermosensitive hydrogel for near-infrared chemo-photothermal therapy on tumor. J. Biomater. Appl..

[86] Yasasvini S., Anusa R.S., VedhaHari B.N., Prabhu P.C., RamyaDevi D. (2017). Topical hydrogel matrix loaded with Simvastatin microparticles for enhanced wound healing activity. Mater. Sci. Eng. C Mater. Biol. Appl..

[87] Caramella C.M., Rossi S., Ferrari F., Bonferoni M.C., Sandri G. (2015). Mucoadhesive and thermogelling systems for vaginal drug delivery. Adv. Drug Deliv. Rev..

[88] Machul A., Mikołajczyk D., Regiel-Futyra A., Heczko P.B., Strus M., Arruebo M., Stochel G., Kyzioł A. (2015). Study on inhibitory activity of chitosan-based materials against biofilm producing *Pseudomonas aeruginosa* strains. J. Biomater. Appl..

[89] Lu Y., Slomberg D.L., Schoenfisch M.H. (2014). Nitric oxide-releasing chitosan oligosaccharides as antibacterial agents. Biomaterials.

[90] Kaye K.S., Pogue J.M. (2015). Infections caused by resistant gram-negative bacteria: Epidemiology and management. Pharmacotherapy.

[91] Dortet L., Boulanger A., Poirel L., Nordmana P. (2014). Bloodstream infections caused by *pseudomonas* spp.: How to detect carbapenemase producers directly from blood cultures. J. Clin. Microbiol..

[92] Kolpen M., Kuhl M., Bjarnsholt T., Hansen C.R., Liengaars L., Kharazmi A., Pressler T., Hoiby N., Jensen P.O. (2014). Nitrous oxide production in sputum from Cystic Fibrosis patients with chronic pseudomonas aeruginosa lung infection. PLoS ONE.

[93] Loveday H.P., Wilson J.A., Kerr K., Pitchers J.T., Walker J. (2014). Association between healthcare water systems and *Pseudomonas aeruginosa* infections: A rapid systematic review. J. Hosp. Infect..

[94] Mesaros N., Nordmann P., Plesiat P., Roussel-Delvallez M., Van Eldere J., Glupczynski Y., Van Laethem Y., Jacobs F., Lebecque P., Malfroot A. (2007). Pseudomonas aeruginosa: Resistance and therapeutic options at the turnof the new millennium. Clin. Microbiol. Infect..

[95] Hetrick E.M., Shin J.H., Stasko N.A., Johnson C.B., Wespe D.A., Holmuhamedov E., Schoenfisch M.H. (2008). Bactericidal Efficacy of Nitric Oxide-Releasing Silica Nanoparticles. ACS Nano.

[96] Barraud N., Schlekeck D., Klebensberger J., Webb J.S., Hasset D.J., Rice S.A., Kjelleberg S. (2009). Nitric oxide signalling in pseudomonas aeruginosa biofilms mediates phosphodiesterase activity, decreased cyclic di-GMP levels and enhanced dispersal. J. Bacteriol..

